# Evaluating the effectiveness of various treatment modalities in vulvar high-grade squamous intraepithelial lesions (vHSIL): a systematic review

**DOI:** 10.1016/j.gore.2026.102038

**Published:** 2026-02-13

**Authors:** Vera J.G.M. Vaessen, Katerina Sidera, Ralf L.O. van de Laar, Heleen J. van Beekhuizen

**Affiliations:** Department of Gynecologic Oncology, Erasmus MC Cancer Institute, University Medical Centre Rotterdam, Dr. Molewaterplein 40, 3015 GD Rotterdam, the Netherlands

**Keywords:** Vulvar High-Grade Squamous Intraepithelial Lesion (vHSIL), Vulvar Intraepithelial Neoplasia (VIN), Human Papillomavirus (HPV), Imiquimod, Excision, Laser ablation, Recurrence

## Abstract

**Objective:**

Vulvar high-grade squamous intraepithelial lesion (vHSIL) is a premalignant condition caused by persistent infection with high-risk human papillomavirus (HPV), with approximately 10% of cases progressing to vulvar carcinoma within ten years. This systematic review evaluates the effectiveness of surgical excision, CO_2_ laser ablation, and imiquimod cream in terms of complete response (CR) and recurrence rates.

**Methods:**

A systematic review was performed following PRISMA guidelines, with literature searches conducted in Medline, Embase, and Web of Science (PROSPERO: CRD42024578702). Eligible studies included adult women (≥18 years) with histopathologically confirmed vHSIL requiring treatment. Two independent reviewers performed study selection and data extraction, and risk of bias was assessed using the Cochrane tools. Due to substantial study heterogeneity, a formal *meta*-analysis was not performed. Instead, pooled CR and recurrence rates were calculated using weighted averages, with 95% confidence intervals. Statistical heterogeneity was assessed with the I^2^ statistic and Chi^2^ test.

**Results:**

26 studies involving 1,705 patients were included. Pooled CR rates ranged from 55 to 100% for excision, 44–85% for CO_2_ laser, and 25–81% for imiquimod. Recurrence rates varied from 0 to 83%, with the lowest recurrence observed in HPV-negative patients treated with imiquimod. Excision resulted in rapid CR but had high recurrence rates, particularly with positive surgical margins. Imiquimod caused local adverse effects (burning, erythema, irritation), but preserved anatomy, as did CO_2_ laser ablation, which lacked histological control.

**Conclusion:**

No single treatment modality proved superior, with all showing high recurrence rates. These results emphasize the importance of individualized treatment strategies and further research to reduce recurrence.

## Highlights

• Systematic review comparing treatments for vulvar intraepithelial neoplasia.
• Evaluates excision, laser ablation, and topical imiquimod therapy.
• Synthesizes global evidence to guide individualized treatment strategies.
• Highlights need for standardized follow-up and long-term outcome reporting.
• Identifies gaps for future research in vulvar precancer management.

## 1 Introduction

Vulvar high-grade squamous intraepithelial lesion (vHSIL) is a precancerous lesion of the vulva, primarily associated with persistent infection by high-risk papillomavirus (HPV) types. The incidence rate of vHSIL has been reported to range between 2.5 and 8.8 cases per 100.000 women per year (Lebreton et al., 2020). Approximately 10% of women diagnosed with vHSIL progress to vulvar cancer within ten years of the initial diagnosis (Ayala et al., 2025, Singh et al., 2023, Thuijs et al., 2021, Classen-von Spee et al., 2024 Jan 8). Classified within the vulvar intraepithelial neoplasia (VIN) spectrum, vHSIL demonstrates its highest prevalence among women aged 30–50 years (Lebreton et al., 2020, Thuijs et al., 2021, Fehr et al., 2013).

Currently, three primary treatment modalities are commonly used for the management of vHSIL. These include surgical excision, laser vaporization, and topical therapies such as imiquimod cream. These treatments aim, firstly, to clear the lesions and relieve the symptoms, and secondly, to prevent progression to invasive vulvar cancer. The optimal treatment can be tailored to everyone's unique situation, considering size and location. Committing to preserving both anatomy and quality of life, including sexual function (Preti et al., 2022).

Despite management of vHSIL using these various therapeutic options (Thuijs et al., 2021, Page et al., 2021 Apr). This recurrence rate leads to repeated interventions, which increase morbidity and have a substantial impact.

There is currently no consensus regarding the optimal management strategy for vHSIL. Each treatment modality presents advantages and limitations. Surgical excision provides histopathological confirmation and high initial clearance rates but is associated with greater anatomical disruption and potential impairment. Laser ablation is mostly less invasive and can preserve anatomical architecture; however, it limits histological verification of complete removal. Topical imiquimod is non-invasive and can be self-administered, although it requires prolonged application and is frequently associated with local invasive inflammatory reactions. Given the therapeutic advantages and limitations of current treatment options, further comparative research is needed to establish the most effective management strategy for vHSIL.

This systematic review aims to evaluate the comparative effectiveness of primary treatment modalities for vHSIL, specifically surgical excision, laser vaporization, and imiquimod cream, in reducing recurrence rates. By synthesizing available data, the review seeks to provide clarity on the most effective treatment, thereby helping to improve patient outcomes and inform clinical practice.

## 2 Methods

This systematic review was performed in accordance with the guidelines outlined in the Preferred Reporting Items for Systematic Reviews and Meta-analyses (PRISMA) Statement (Page et al., 2021 Apr). The protocol was registered in PROSPERO on September 16th, 2024, with registration number CRD42024578702.

### 2.1 Inclusion and exclusion criteria

This systematic review follows a PICO/PEO framework to ensure a structured approach. The population included women aged 18 years or older with histopathologically confirmed vHSIL. Eligible study designs included randomized controlled trials (RCTs), cohort studies, case-control studies, systematic reviews, and case series. Case reports, editorials, ongoing trials, preprints, and commentaries were excluded. Studies evaluating primary treatment modalities for vHSIL, such as surgical excision, CO2 laser vaporization, topical imiquimod cream or other recognized treatments, were included. Studies focusing on prophylactic or therapeutic HPV vaccines, watchful waiting (no-treatment) or experimental therapies were excluded. Only studies published in the English language were considered eligible to ensure uniformity in data interpretation and methodological appraisal. Outcomes of interest were recurrence rates, defined as the reappearance or persistence of vHSIL after 6 months after treatment initiation, rates of complete and partial response, residual disease, along with any treatment-related adverse effects, and the impact of treatments on quality of life.

### 2.2 Search strategies

The search strategy was developed by the corresponding authors (V.V. and K.S.) in collaboration with a medical information specialist at the Erasmus Medical Center library. The search used Medical Subject Headings (MeSH) and keywords related to vHSIL and its treatment modalities. After the original search was performed on July 5th, 2024, the search was last updated on July 14th, 2025, across Medline ALL (Ovid), Embase (Embase.com), and Web of Science Core Collection (Web of Knowledge) (Appendix A) and covered the years from the inception of each database to the present. Additional studies were identified through a snowballing method, which entailed systematically reviewing the reference lists of included articles and tracking their subsequent citations. The search was updated on July 14th, 2025, to identify any additional relevant articles published in the interim.

### 2.3 Data extraction and Risk of Bias assessment

Title and abstract screening for eligibility were independently performed by two reviewers (V.V. and K.S.) using the Covidence systematic review platform (Babineau, 2014 Aug 1). Potentially relevant articles were subsequently retrieved for full-texts assessment. Any disagreements during the screening or selection process were resolved through discussion with a third independent reviewer (R.L.).

Data extraction was conducted independently by the same two reviewers (V.V. and K.S.) using a customized standardized form within Covidence. Extracted data included author(s), year, country, study period, study structure, population characteristics (e.g. sample size, age), treatments (e.g. surgical excision, laser vaporization, topical therapy), follow-up duration, and study objectives. Outcomes of interest were recorded using measurement scales, including percentages, relative risks (RRs), odds ratios (ORs), 95% confidence intervals (CIs) were applicable, and other relevant outcome measures. Missing data were marked with a dash when attempts to contact the study author for clarification were unsuccessful. Consensus on the extracted items was achieved through discussion before commencing data analysis.

Risk of bias was independently assessed by the same two reviewers (V.V. and K.S.) For non-randomized studies, the Cochrane Risk of Bias in Non-randomized Studies of Interventions (ROBINS-I V2) tool was used, while randomized controlled trials were evaluated using the Cochrane Risk of Bias 2 (RoB 2) tool (Sterne et al., 2016 Oct, Sterne et al., 2019 Aug). Risk of bias assessments were visualized using the robvis tool (McGuinness and Higgins, 2021) (Fig. 4 and Fig. 5). Any discrepancies between reviewers were solved through consensus. Following ROBINS-I guidelines, studies rated as having a “critical” risk of bias, specifically those that failed to control for confounding, were excluded from further analysis. These studies lacked adequate statistical methods for key prognostic variables, limiting the validity of their causal interpretations.

### 2.4 Statistical analyses

All included studies were summarized in a comprehensive supplementary table, reporting study design, patient characteristics, treatment regimens, eligibility criteria, primary and secondary outcomes, adverse events, and authors’ conclusions (Supplement).

Due to substantial clinical and methodological heterogeneity, no formal *meta*-analysis of dichotomous outcomes was performed. Instead, data were summarized narratively, with pooled complete response (CR) and recurrence rates calculated for each treatment modality (imiquimod 5%, CO2 laser ablation, and surgical excision). The pooled CR and recurrence rates were computed using weighted averages, considering the sample size and outcomes reported in each study.

Forest plots were created to visually summarize the proportion of patients achieving a complete response as well as the recurrence rate for the treatments assessed. A random-effects model was used to account for expected clinical and methodological heterogeneity.

Results are reported as pooled proportions with 95% CI. Statistical heterogeneity was assessed with the I^2^ statistic and the Chi^2^ (Q) test, with I^2^ values greater than 50% considered to indicate substantial heterogeneity.

All analyses were conducted in R Studio (version 4.4.1) using the *meta* and *metafor* packages (Balduzzi et al., 2019 Nov).

## 3 Results

**Fig. 1:**
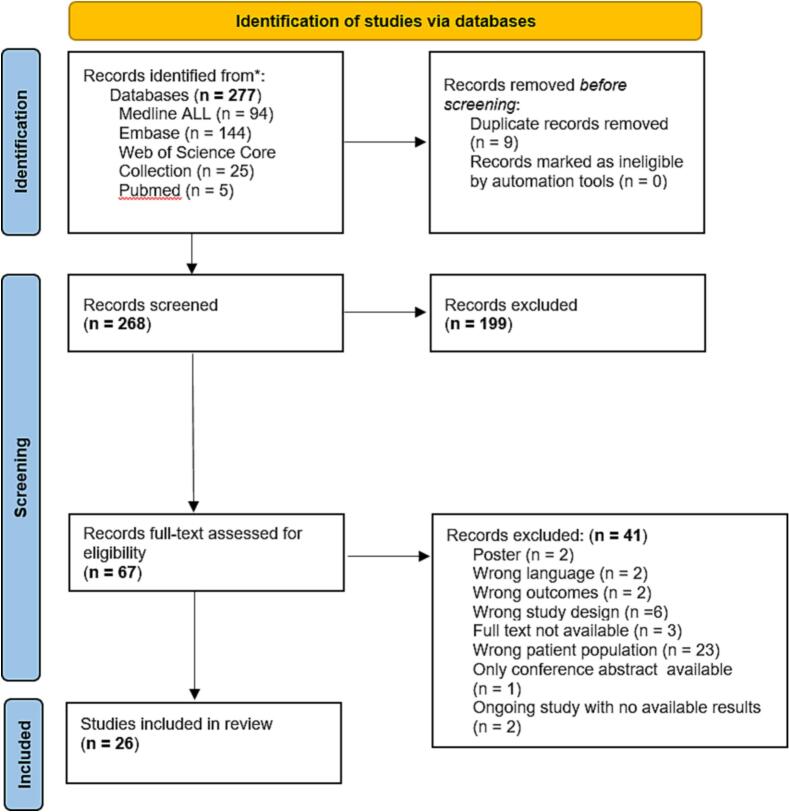
PRISMA 2020 flow diagram.

A total of 268 articles were screened based on title and abstract. Following the removal of duplicates and further screening of full texts, 26 studies met the eligibility criteria and were included in the systematic review. The study selection process is detailed in the PRISMA flow diagram (Fig. 1).

Several studies that appeared relevant during title and abstract screening were excluded after full-text review for not meeting the eligibility criteria. For example, articles combining vulvar and vaginal intraepithelial neoplasia without separate outcome reporting were excluded (6). Another study was a narrative review lacking original clinical outcome data (Daayana et al., 2011). Bassetty et al. focused primarily on vulvectomy and included patients with invasive vulvar cancer (Bassetty et al., 2022). Boonlikit et al. reported combined outcomes for vulvar and vaginal lesions rather than isolated vulvar HSIL (Boonlikit and Tangterdchanakit, 2024).

The included studies were conducted between 2000 and 2024 in 17 different countries.

Of the 26 included studies, six were randomized controlled trials (RCTs), six were prospective cohort studies, ten were retrospective cohort studies, three were prospective case series, and one was a Simon two-stage study (Table 1). Altogether, the studies encompassed 1,705 women diagnosed with vHSIL. Sample sizes varied considerably, ranging from 8 patients in the smallest study (Marchitelli et al., 2004) to 405 in the largest (Jones et al., 2005). The included studies evaluated a range of treatment modalities. Sixteen studies assessed 5% imiquimod cream as the primary intervention (Marchitelli et al., 2004, Wendling J, Saiag P, Berville-Levy S, Bourgault-Villada I, Clerici T, Moyal-Barracco M. Treatment of Undifferentiated Vulvar Intraepithelial Neoplasia With 5% Imiquimod Cream: A Prospective Study of 12 Cases. Arch Dermatol [Internet]., 2004, Bruchim et al., 2007, Le et al., 2007, Mathiesen et al., 2007 Nov, Van Seters et al., 2008, Terlou et al., 2011, Frega et al., 2013, Van Esch et al., 2013, Westermann et al., 2013 Mar, Tristram et al., 2014 Nov, Kim et al., 2015, De Figueiredo et al., 2017, Fernández-Montolí et al., 2022, Trutnovsky et al., 2022, Xavier et al., 2023, Trutnovsky et al., 2024), while sixteen reported on surgical techniques, including cold knife excision or CO2 laser vaporization (Jones et al., 2005, Bruchim et al., 2007, Frega et al., 2013, Frega et al., 2013, Van Esch et al., 2013, De Figueiredo et al., 2017, Trutnovsky et al., 2022, Xavier et al., 2023, Trutnovsky et al., 2024, Thuis et al., 2000, Ribeiro et al., 2012, Leufflen et al., 2013, Gentile et al., 2014, Bianchi et al., 2022, Beavis et al., 2023, Zhou et al., 2023 Dec). Among these, three studies reported the detection of invasive disease on final pathology reports following excisional procedures. Trutnovsky et al. (2022) (Trutnovsky et al., 2022) identified invasive disease in 4 patients (8%) who underwent primary surgical treatment, while Xavier et al. reported invasive carcinoma in 4 patients (11.8%) (Xavier et al., 2023). Similarly, Thuis et al. (Thuis et al., 2000) identified occult invasive carcinoma in 6 patients (15%) following surgical excision.

Progression to invasive vulvar carcinoma following CO2 laser ablation was reported in six studies (Bruchim et al., 2007, Van Esch et al., 2013, De Figueiredo et al., 2017, Bianchi et al., 2022, Beavis et al., 2023, Frega et al., 2011). Two studies observed no progression to invasive disease during follow-up after laser treatment (Bruchim et al., 2007, De Figueiredo et al., 2017). In contrast, other studies reported invasive recurrence after laser treatment. Van Esch et al. (Van Esch et al., 2013) reported progression to vulvar carcinoma in 15.1% of patients treated with laser, excision, or combined approaches. Frega et al. (Frega et al., 2013) reported one case (1%) of invasive vulvar carcinoma after CO2 laser excision, while Bianchi et al. (Bianchi et al., 2022) observed progression to vulvar squamous cell carcinoma in one patient (2%) in the CO2 laser excision group. Additionally, Beavis et al. (Beavis et al., 2023) reported progression to invasive squamous cell carcinoma in 5 patients (12.2%) following vHSIL recurrence, including 2 patients (9.5%) in the CO2 laser ablation group and 3 patients (14.3%) in the plasma energy ablation group.

Less commonly reported treatment modalities included photodynamic therapy (Zhou et al., 2023 Dec), cidofovir 1% gel (Tristram et al., 2014 Nov), and plasma energy ablation (Beavis et al., 2023). Two studies evaluated combination treatments, typically surgical excision followed by imiquimod (Xavier et al., 2023, Gentile et al., 2014). Treatment regimens varied, with imiquimod typically applied two to three times weekly over 12 to 24 weeks (Le et al., 2007, Mathiesen et al., 2007 Nov, Van Seters et al., 2008, Tristram et al., 2014 Nov). Follow-up durations ranged widely from 6 months to over 7 years (Terlou et al., 2011, Frega et al., 2013, Van Esch et al., 2013, Fernández-Montolí et al., 2022).

Reported complete response (CR) rates differed between treatment modalities and studies. For imiquimod, CR rates ranged from 25% to 81%, with the highest rates observed in randomized trials such as Mathiesen (2007) (Mathiesen et al., 2007 Nov), van Seters (2008) (Van Seters et al., 2008), and Trutnovsky (2022) (Trutnovsky et al., 2022), who reported CR rates around 70% to 80% at six months. Surgical excision demonstrated CR rates between 55% and 100%, as described by Frega (2013) (Frega et al., 2013) and Leufflen (2013) (Leufflen et al., 2013), though recurrence remained frequent during long-term follow-up. CO_2_ laser ablation achieved CR rates between 44% and 85%, as reported by Bruchim (2007) (Bruchim et al., 2007) and Leufflen (2013) (Leufflen et al., 2013). Photodynamic therapy (Zhou et al., 2023 Dec) demonstrated a CR of 91% while cidofovir (Tristram et al., 2014 Nov) demonstrated a CR of 46%.

Recurrence rates also varied substantially across treatments. Among patients treated with imiquimod, recurrence rates ranged from 0% to 83%, with lower recurrence generally reported in patients who achieved HPV clearance (Terlou et al., 2011, Fernández-Montolí et al., 2022). Surgical excision studies reported recurrence rates ranging from 8% to 81% (Frega et al., 2013, Leufflen et al., 2013), with higher rates observed in cases with positive surgical margins. Recurrence rates following laser ablation ranged from 27% to 56%. Progression to vulvar carcinoma was observed in up to 15% of patients overall, with higher progression rates seen in immunocompromised individuals and those with multifocal disease (Van Esch et al., 2013, Xavier et al., 2023).

Adverse events were commonly reported across treatment modalities. Local side effects such as burning, erythema, and irritation were frequently reported with imiquimod, leading to treatment discontinuation in 8% to 18% of cases (Westermann et al., 2013 Mar, Fernández-Montolí et al., 2022). Surgical excision and laser treatment were associated with postoperative pain, scarring, and vulvar anatomical changes, although serious complications were rare (Leufflen et al., 2013, Bianchi et al., 2022). Photodynamic therapy was generally well tolerated, with only mild irritation reported (Zhou et al., 2023 Dec). Plasma energy ablation appeared to have a safety profile comparable to CO_2_ laser ablation, with few complications reported (Beavis et al., 2023).

Other clinical outcomes included quality of life and psychosexual distress, which were evaluated in two studies (Van Seters et al., 2008, Trutnovsky et al., 2024). These studies found that both surgical and non-surgical treatments were associated with stable psychosexual functioning and acceptable aesthetic outcomes over time. Notably, Trutnovsky (2024) found no differences in psychosexual distress or aesthetic satisfaction between patients treated with imiquimod and those treated surgically (Trutnovsky et al., 2024). Several studies have highlighted the importance of HPV clearance as a marker of treatment success and a predictor of lower recurrence rates (Van Seters et al., 2008, Fernández-Montolí et al., 2022).

**Fig. 2:**
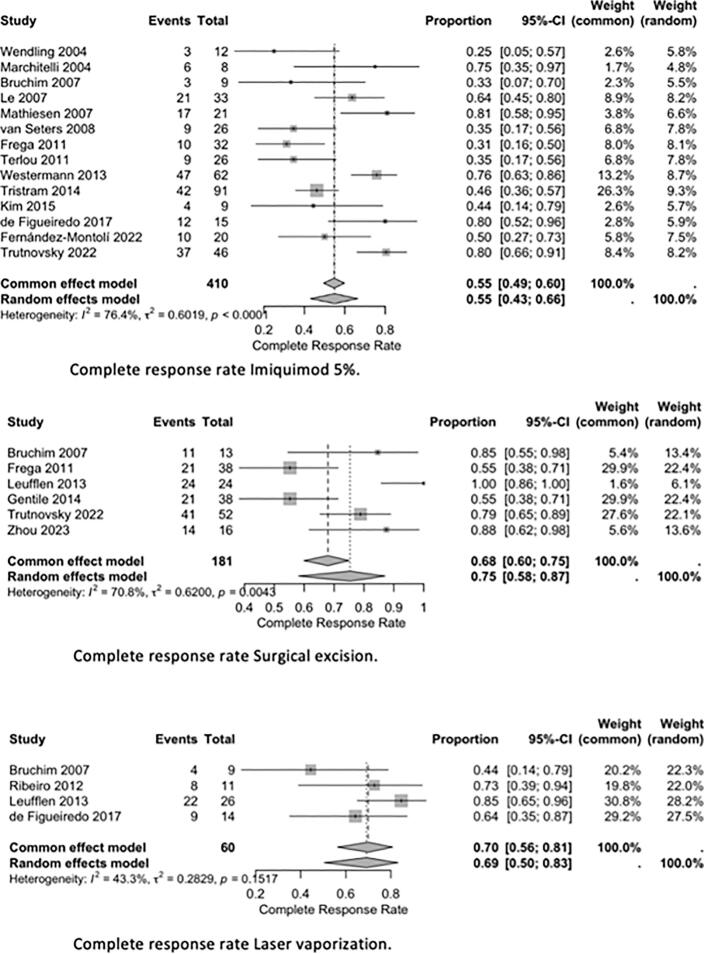
Complete response rate lmiquimod 5%. Complete response rate Surgical excision. Complete response rate Laser vaporization.

**Fig. 3:**
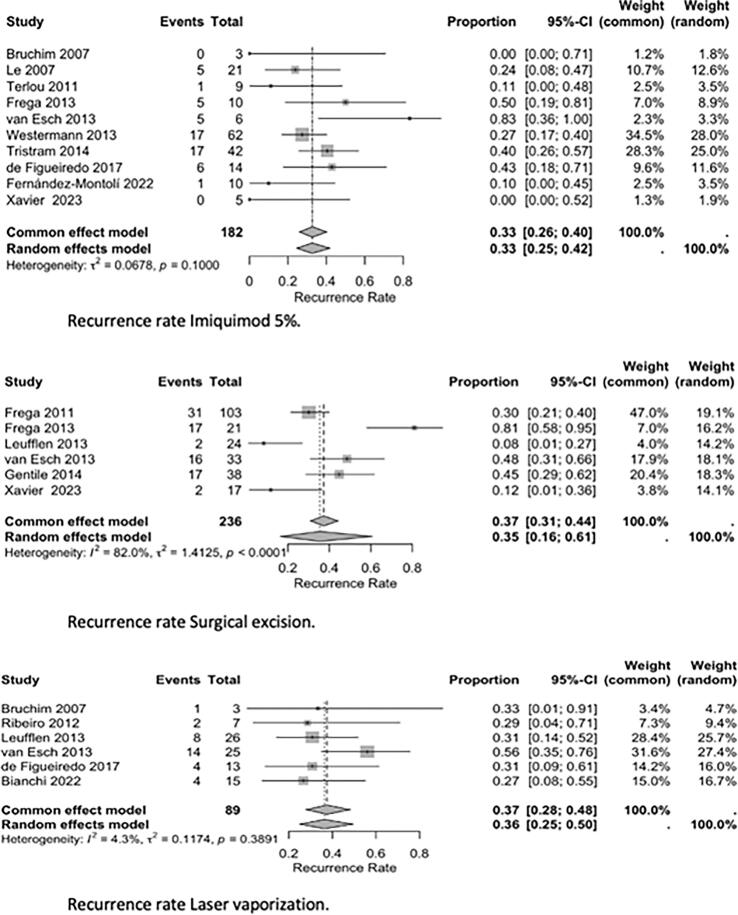
Recurrence rate lmiquimod 5%. Recurrence rate Surgical excision. Recurrence rate Laser vaporization.

Forest plots (Fig. 2, Fig. 3) were created to visually summarize the proportion of patients achieving a complete response as well as recurrence rate across studies evaluating imiquimod 5%, CO2 laser vaporization and surgical excision (cold knife excision). The pooled complete response rate for imiquimod was 55% (95% CI: 43–66%). Due to differences in study design and patient selection there was considerable heterogeneity. For surgical excision, the pooled complete response rate was higher at 75% (95% CI: 58–87%). This variability was largely attributed to differences in surgical margin status, and follow-up duration across studies. Laser vaporization achieved a pooled complete response rate of 69% (95% CI: 56–83%), with heterogeneity reflecting variation in definitions of recurrence, and follow-up duration across studies. The pooled recurrence rate for imiquimod was 35% (95% CI: 26–45%), again with notable heterogeneity influenced by follow-up length and definitions of recurrence. For surgical excision, the pooled recurrence rate was 36% (95% CI: 16–61%), with wide confidence intervals and significant heterogeneity caused by differences in surgical margins and follow-up duration.

**Fig. 4:**
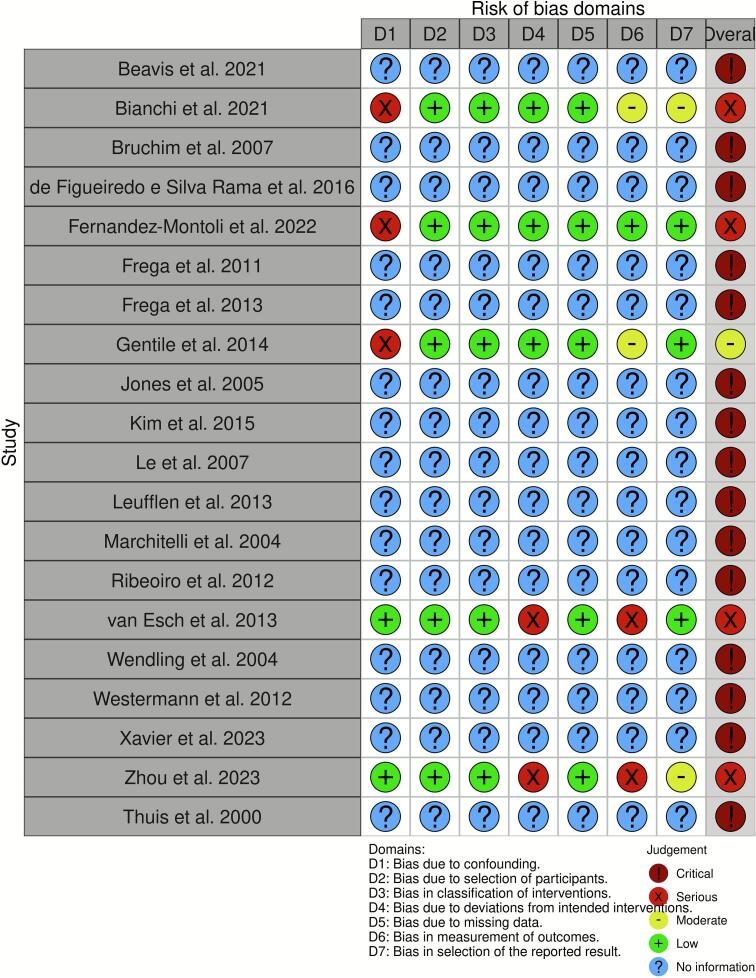

**Fig. 5:**
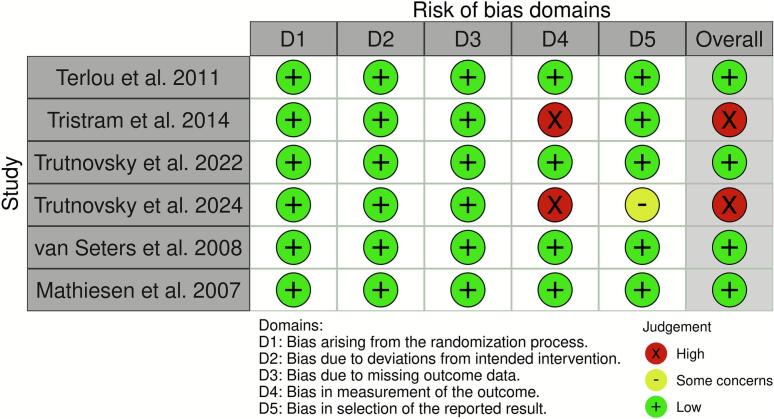

This heterogeneity affects the interpretation of the pooled rates, as these estimates represent averages from methodologically diverse studies, which limits their interpretability and generalizability. The variation in study methodologies, including differences in surgical approaches, recurrence definitions, and follow-up durations, underscores the need for careful consideration when comparing these pooled rates across studies.

## 4 Discussion

### 4.1 Summary of Main Results

This systematic review compared the effectiveness of imiquimod 5%, surgical excision, and laser vaporization for the treatment of vHSIL. Our findings confirm that all three modalities achieve varying rates of complete response, but recurrence around 36% occurs regardless of the treatment of choice. These results along with existing literature supports the need for long-term follow-up, regardless of initial treatment modality. Our findings align with and extend the conclusions of prior systematic reviews on the management of vulvar HSIL. Pepas et al. (2015), synthesizing only randomized controlled trials of medical therapy, confirmed that topical imiquimod achieves substantially higher clearance rates than placebo and performs similarly to cidofovir, albeit with frequent local inflammatory adverse effects (Pepas et al., 2015). However, that review did not compare medical and surgical modalities, and long-term outcomes remained unclear. Lawrie et al. (2016) found that both surgical excision and CO_2_ laser have similar effectiveness, but high recurrence rates persist with either modality. In that analysis, imiquimod and cidofovir were superior to placebo in inducing short-term regression. Still, uncertainty persisted regarding the durability of response and the relative role of medical versus surgical treatment (Lawrie et al., 2016). Simões et al. (2025) limited their *meta*-analysis to randomized trials and found that imiquimod significantly was more effective than placebo and non-inferior to surgery for short-term response and recurrence. They suggest imiquimod as a first-line alternative for select women. Their findings align with our supplementary meta-analysis of two placebo-controlled RCTs (Mathiesen et al., 2007 Nov, Van Seters et al., 2008) (Supplementary figure 1). The pooled relative risk for complete response was markedly higher for imiquimod than for placebo, mirroring the 55% overall response observed in our main analysis. Our review, which includes both randomized and observational studies, examines a broader evidence base (Simões et al., 2025).

### 4.2 Strengths and Weakness

The main strengths of this study are the comprehensive screening of the available literature and the quality assessment, both of which enhance the reliability of the synthesized evidence. Despite the heterogeneity of the included studies, this systematic review synthesizes current evidence on the effectiveness of treatment modalities for vHSIL.

Several limitations should be acknowledged. Considerable heterogeneity in both clinical and methodological aspects, together with a high risk of bias across included studies, reduces the robustness of the conclusions. The relatively small number of included studies, most of which were retrospective in nature, introduces additional bias. Moreover, the evidence base is predominantly derived from Western populations, limiting generalizability to other settings. None of the studies provided data on immunocompromised patients, who are likely to represent a group at higher risk (Tanaka et al., 2016 Aug, Liu et al., 2018 Mar 27, Wielgos et al., 2022). Variation in follow-up durations and a wide variety of treatment regimens further complicate the interpretation and comparability of the outcomes. Furthermore, HPV status was not consistently documented across cohorts, which limits the role in treatment response and recurrence. Our findings demonstrate the critical role of HPV clearance in predicting recurrence after treatment for vHSIL, with patients who cleared HPV following imiquimod treatment having lower recurrence rates. These results align with the previous study of Seters et.al (Van Seters et al., 2008), which demonstrated a strong link between HPV clearance and histologic regression. These results highlight the importance of HPV clearance as a key factor in evaluating treatment success and suggest that monitoring HPV status during follow-up could guide decisions on additional interventions to prevent recurrence. However, the inconsistent reporting of HPV status emphasizes the need for more standardizing HPV testing in future studies.

Finally, treatment with imiquimod was associated with high dropout rates, leading to incomplete treatment courses and potential underestimation of its actual effectiveness.

### 4.3 Implications for Practice and Future Research

This study provides a comprehensive synthesis to date on the effectiveness of treatment modalities for patients with vHSIL. By comparing imiquimod 5%, surgical excision, and laser vaporization, we demonstrate that all three treatments achieve varying rates of complete response, yet recurrence occurs irrespective of the chosen modality. The findings confirm that vulvar HSIL demonstrates variable recurrence rates across different treatment modalities, highlighting the importance of long-term follow-up.

From a clinical perspective, our results emphasize the importance of HPV clearance as a predictor for recurrence and treatment success. Clinicians should consider incorporating HPV testing as a component of post-treatment monitoring, given its potential role in guiding therapeutic decision-making and risk stratification. Achieving HPV clearance post-treatment is likely to reduce the risk of recurrence. Furthermore, given the significant dropout rates associated with imiquimod 5%, leading to incomplete treatment courses in some cases, it is crucial to address patient adherence and ensure comprehensive follow-up to avoid underestimating the treatment’s true efficacy.

However, the data are limited and heterogeneous, highlighting the urgent need for more robust evidence. Future research should focus specifically on well-designed prospective studies with larger sample sizes that compare surgical and medical treatments, with standardized follow-up, consistent reporting of HPV status, and clear documentation of whether immunocompromised women are represented in the study population. Ultimately this will improve decision-making for the best treatment for patients with vHSIL.

## CRediT authorship contribution statement

**Vera J.G.M. Vaessen:** Writing – review & editing, Writing – original draft, Visualization, Validation, Methodology, Formal analysis, Data curation, Conceptualization. **Katerina Sidera:** Writing – review & editing, Writing – original draft, Visualization, Validation, Methodology, Formal analysis, Data curation, Conceptualization. **Ralf L.O. van de Laar:** Writing – review & editing. **Heleen J. van Beekhuizen:** Writing – review & editing.

## Declaration of competing interest

The authors declare that they have no known competing financial interests or personal relationships that could have appeared to influence the work reported in this paper.

## References

[b0005] Lebreton M., Carton I., Brousse S., Lavoué V., Body G., Levêque J. (2020). Vulvar intraepithelial neoplasia: Classification, epidemiology, diagnosis, and management. J Gynecol Obstet Hum Reprod [internet]..

[b0010] Ayala M, Fatehi M. Vulvar Intraepithelial Neoplasia. In: StatPearls [Internet]. Treasure Island (FL): StatPearls Publishing; 2025 [cited 2025 Sept 25]. Available from: http://www.ncbi.nlm.nih.gov/books/NBK540982/. 31082026

[b0015] Singh D., Vignat J., Lorenzoni V., Eslahi M., Ginsburg O., Lauby-Secretan B. (2023). Global estimates of incidence and mortality of cervical cancer in 2020: a baseline analysis of the WHO Global Cervical Cancer Elimination Initiative. Lancet Glob. Health.

[b0020] Thuijs N.B., van Beurden M., Bruggink A.H., Steenbergen R.D.M., Berkhof J., Bleeker M.C.G. (2021). Vulvar intraepithelial neoplasia: Incidence and long-term risk of vulvar squamous cell carcinoma. Int. J. Cancer.

[b0025] Classen-von Spee S., Baransi S., Fix N., Rawert F., Luengas-Würzinger V., Lippert R. (2024 Jan 8). Pelvic Exenteration for Recurrent Vulvar Cancer: a Retrospective Study. Cancers.

[b0030] Fehr M.K., Baumann M., Mueller M., Fink D., Heinzl S., Imesch P. (2013). Disease progression and recurrence in women treated for vulvovaginal intraepithelial neoplasia. J. Gynecol. Oncol..

[b0035] Preti M., Joura E., Vieira-Baptista P., Van Beurden M., Bevilacqua F., Bleeker M.C.G. (2022). The European Society of Gynaecological Oncology (ESGO), the International Society for the Study of Vulvovaginal Disease (ISSVD), the European College for the Study of Vulval Disease (ECSVD) and the European Federation for Colposcopy (EFC) Consensus statements on Pre-invasive Vulvar Lesions. J. Low. Genit. Tract Dis..

[b0040] Page M.J., McKenzie J.E., Bossuyt P.M., Boutron I., Hoffmann T.C., Mulrow C.D. (2021 Apr). The PRISMA 2020 statement: an updated guideline for reporting systematic reviews. Int J Surg Lond Engl..

[b0045] Babineau J. (2014 Aug 1). Product Review: Covidence (Systematic Review Software). J Can Health Libr Assoc J Assoc Bibl Santé Can..

[b0050] Sterne J.A., Hernán M.A., Reeves B.C., Savović J., Berkman N.D., Viswanathan M. (2016 Oct). ROBINS-I: a tool for assessing risk of bias in non-randomised studies of interventions. BMJ.

[b0055] Sterne J.A.C., Savović J., Page M.J., Elbers R.G., Blencowe N.S., Boutron I. (2019 Aug). RoB 2: a revised tool for assessing risk of bias in randomised trials. BMJ.

[b0060] McGuinness L.A., Higgins J.P.T. (2021 Jan). Risk-of-bias VISualization (robvis): an R package and Shiny web app for visualizing risk-of-bias assessments. Res. Synth. Methods.

[b0065] Balduzzi S., Rücker G., Schwarzer G. (2019 Nov). How to perform a meta-analysis with R: a practical tutorial. Evid. Based Ment. Health.

[b0070] Daayana S., Winters U., Stern P.L., Kitchener H.C. (2011). Clinical and immunological response to photodynamic therapy in the treatment of vulval intraepithelial neoplasia. Photochem. Photobiol. Sci..

[b0075] Bassetty KC, Thomas A, Chandy RG, Thomas DS, Thomas V, Peedicayil A, et al. Vulval Intraepithelial Neoplasia 3: A Clinico-Pathological Review in a Tertiary Care Centre Over 10 Years. J Obstet Gynecol India. 2022;72((Bassetty K.C.; Thomas A.; Chandy R.G.; Thomas D.S.; Thomas V.; Peedicayil A.; Sebastian A., sebastian.ajit@gcom) Department of Gynaecologic Oncology, Christian Medical College, Vellore, India):334–9. 10.1007/s13224-022-01659-2 PMC9343544 35928075

[b0080] Boonlikit S., Tangterdchanakit P. (2024). Multicentricity and the risk of Recurrence/Persistence after Laser Vaporization for High-Grade Vulvar and Vaginal Intraepithelial Neoplasia. World J Oncol..

[b0085] Marchitelli C., Secco G., Perrotta M., Lugones L., Pesce R., Testa R. (2004). Treatment of bowenoid and basaloid vulvar intraepithelial neoplasia 2/3 with imiquimod 5% Cream. J Reprod Med Obstet Gynecol..

[b0090] Jones R.W., Rowan D.M., Stewart A.W. (2005). Vulvar intraepithelial neoplasia: Aspects of the natural history and outcome in 405 women. Obstet. Gynecol..

[b0095] Wendling J, Saiag P, Berville-Levy S, Bourgault-Villada I, Clerici T, Moyal-Barracco M. Treatment of Undifferentiated Vulvar Intraepithelial Neoplasia With 5% Imiquimod Cream: A Prospective Study of 12 Cases. Arch Dermatol [Internet]. 2004 Oct 1 [cited 2025 Oct 14];140(10). Available from: http://archderm.jamanetwork.com/article.aspx?doi=10.1001/archderm.140.10.1220. 10.1001/archderm.140.10.1220 15492184

[b0100] Bruchim I., Gotlieb W.H., Mahmud S., Tunitsky E., Grzywacz K., Ferenczy A. (2007). HPV-related vulvar intraepithelial neoplasia: Outcome of different management modalities. Int. J. Gynecol. Obstet..

[b0105] Le T., Menard C., Hicks-Boucher W., Hopkins L., Weberpals J., Fung-Kee-Fung M. (2007). Final results of a phase 2 study using continuous 5% Imiquimod cream application in the primary treatment of high-grade vulva intraepithelial neoplasia. Gynecol. Oncol..

[b0110] Mathiesen O., Buus S., Cramers M. (2007 Nov). Topical imiquimod can reverse vulvar intraepithelial neoplasia: a randomised, double-blinded study. Gynecol. Oncol..

[b0115] Van Seters M., Van Beurden M., Ten Kate F.J.W., Beckmann I., Ewing P.C., Eijkemans M.J.C. (2008). Treatment of vulvar intraepithelial neoplasia with topical imiquimod. N. Engl. J. Med..

[b0120] Terlou A., Van Seters M., Ewing P.C., Aaronson N.K., Gundy C.M., Heijmans-Antonissen C. (2011). Treatment of vulvar intraepithelial neoplasia with topical imiquimod: Seven years median follow-up of a randomized clinical trial. Gynecol. Oncol..

[b0125] Frega A., Sesti F., Sopracordevole F., Biamonti A., Scirpa P., Milazzo G.N. (2013). Imiquimod 5% cream versus cold knife excision for treatment of VIN 2/3: a five-year follow-up. Eur. Rev. Med. Pharmacol. Sci..

[b0130] Van Esch E.M.G., Dam M.C.I., Osse M.E.M., Putter H., Trimbos B.J.B.M.Z., Fleuren G. (2013). Clinical characteristics associated with development of recurrence and progression in usual-type vulvar intraepithelial neoplasia. Int. J. Gynecol. Cancer.

[b0135] Westermann C., Fischer A., Clad A. (2013 Mar). Treatment of vulvar intraepithelial neoplasia with topical 5% imiquimod cream. Int. J. Gynecol. Obstet..

[b0140] Tristram A., Hurt C.N., Madden T., Powell N., Man S., Hibbitts S. (2014 Nov). Activity, safety, and feasibility of cidofovir and imiquimod for treatment of vulval intraepithelial neoplasia (RT3VIN): a multicentre, open-label, randomised, phase 2 trial. Lancet Oncol..

[b0145] Kim J.M., Lee H.J., Kim S.H., Kim H.S., Ko H.C., Kim B.S. (2015). Efficacy of 5% Imiquimod Cream on Vulvar Intraepithelial Neoplasia in Korea: pilot Study. Ann. Dermatol..

[b0150] De Figueiredo E., Silva Rama A.L., De Gois Speck N.M., De Carvalho C.R.N., Schimidt M.A., Ribalta J.C.L. (2017). Imiquimod cream and CO2 laser vaporization in vulvar intraepithelial neoplasia (VIN) 2/3 treatment. Eur. J. Gynaecol. Oncol..

[b0155] Fernández-Montolí M.E., Heydari F., Lavecchia F., Pavón M.Â., Guerra E., Matias-Guiu X. (2022). Vulvar High-Grade Squamous Intraepithelial Lesions Treated with Imiquimod: can Persistence of Human Papillomavirus Predict Recurrence?. Cancers.

[b0160] Trutnovsky G., Reich O., Joura E.A., Holter M., Ciresa-König A., Widschwendter A. (2022). Topical imiquimod versus surgery for vulvar intraepithelial neoplasia: a multicentre, randomised, phase 3, non-inferiority trial. Lancet.

[b0165] Xavier J., Figueiredo R., Vieira-Baptista P. (2023). Vulvar High-Grade Squamous Intraepithelial Lesion and the risk of Recurrence and Progression to Cancer. J. Low. Genit. Tract Dis..

[b0170] Trutnovsky G., Holter M., Gold D., Kopera D., Deban J., Misut D. (2024). Aesthetic Outcome and Psychosexual Distress after Treatment for Vulvar High-Grade Squamous Intraepithelial Lesions. J. Low. Genit. Tract Dis..

[b0175] Thuis Y.N., Campion M., Fox H., Hacker N.F. (2000). Contemporary experience with the management of vulvar intraepithelial neoplasia. Int. J. Gynecol. Cancer.

[b0180] Ribeiro F., Figueiredo A., Paula T., Borrego J. (2012). Vulvar intraepithelial neoplasia: Evaluation of treatment modalities. J. Low. Genit. Tract Dis..

[b0185] Leufflen L., Baermann P., Rauch P., Routiot T., Bezdetnava L., Guillemin F. (2013). Treatment of vulvar intraepithelial neoplasia with CO2 laser vaporization and excision surgery. J. Low. Genit. Tract Dis..

[b0190] Gentile M., Bianchi P., Sesti F., Sopracordevole F., Biamonti A., Scirpa P. (2014). Adjuvant topical treatment with imiquimod 5% after excisional surgery for VIN 2/3. Eur. Rev. Med. Pharmacol. Sci..

[b0195] Bianchi C., Auzzi N., Turrini I., De Magnis A., Fallani M.G., Fambrini M. (2022). CO2 laser colposcopic guided surgery for the see and treat management of VHSIL: a preliminary experience. Lasers Med. Sci..

[b0200] Beavis A, Najjar O, Murdock T, Abing A, Fader A, Wethington S, et al. Treatment of vulvar and vaginal dysplasia: Plasma energy ablation versus carbon dioxide laser ablation. Int J Gynecol Cancer [Internet]. 2023;((Beavis A., abeavis2@jhmi.edu; Najjar O.; Fader A.; Wethington S.; Stone R.; Ferriss J.S.; Levinson K.) Department of Gynecology and Obstetrics, Johns Hopkins School of Medicine, Baltimore, MD, United States). Available from: https://www.embase.com/search/results?subaction=viewrecord&id=L2024115566&from=export. 10.1136/ijgc-2021-002913 PMC9415775 34610972

[b0205] Zhou M., Su Y., Tong Y., Zhang C., Yuan S., Zhang M. (2023 Dec). Comparative study of topical 5-aminolevulinic acid photodynamic therapy and surgery for the treatment of vulvar squamous intraepithelial lesion. Photodiagn. Photodyn. Ther..

[b0210] Frega A., Sopracordevole F., Scirpa P., Biamonti A., Lorenzon L., Scarani S. (2011). The re-infection rate of high-risk HPV and the recurrence rate of vulvar intraepithelial neoplasia (VIN) usual type after surgical treatment. Med. Sci. Monit..

[b0215] Pepas L, Kaushik S, Nordin A, Bryant A, Lawrie TA. Medical interventions for high-grade vulval intraepithelial neoplasia. Cochrane Gynaecological, Neuro-oncology and Orphan Cancer Group, editor. Cochrane Database Syst Rev [Internet]. 2015 Aug 18 [cited 2025 July 7];2016(9). Available from: http://doi.wiley.com/10.1002/14651858.CD007924.pub3. 10.1002/14651858.CD007924.pub3 PMC6457779 26284429

[b0220] Lawrie TA, Nordin A, Chakrabarti M, Bryant A, Kaushik S, Pepas L. Medical and surgical interventions for the treatment of usual-type vulval intraepithelial neoplasia. Cochrane Gynaecological, Neuro-oncology and Orphan Cancer Group, editor. Cochrane Database Syst Rev [Internet]. 2016 Jan 5 [cited 2025 July 7];2016(9). Available from: http://doi.wiley.com/10.1002/14651858.CD011837.pub2. 10.1002/14651858.CD011837.pub2 PMC6457805 26728940

[b0225] Simões AC, Sarmento AC, Aquino AC, Eleutério-Jr J, Do Val Guimarães IC, Falsetta ML, et al. Treatment Interventions for Usual-Type Vulvar Intraepithelial Neoplasia: A Systematic Review and Meta-analysis. J Low Genit Tract Dis [Internet]. 2025 June 6 [cited 2025 July 7]; Available from: https://journals.lww.com/10.1097/LGT.0000000000000901. 10.1097/LGT.0000000000000901 40476854

[b0230] Tanaka Y., Ueda Y., Kakuda M., Kubota S., Matsuzaki S., Nakagawa S. (2016 Aug). Clinical outcomes of abnormal cervical cytology and human papillomavirus-related lesions in patients with organ transplantation: 11-year experience at a single institution. Int. J. Clin. Oncol..

[b0235] Liu G., Sharma M., Tan N., Barnabas R.V. (2018 Mar 27). HIV-positive women have higher risk of human papilloma virus infection, precancerous lesions, and cervical cancer. AIDS.

[b0240] Wielgos A., Pietrzak B., Suchonska B., Sikora M., Rudnicka L., Wielgos M. (2022 Mar 16). A Six-Year Gynecological Follow-up of Immunosuppressed Women with a High-Risk Human Papillomavirus Infection. Int. J. Environ. Res. Public Health.

