# Supplementary material for: Evaluating the effectiveness of various treatment modalities in vulvar high-grade squamous intraepithelial lesions (vHSIL): a systematic review
Source: Gynecol Oncol Rep. 2026 Feb 13;64:102038. doi: 10.1016/j.gore.2026.102038 (PMC12925432; doi:10.1016/j.gore.2026.102038)
Supplement: Supplementary Data 1 [file mmc1.docx]

**Appendix A. Search strategy**

| **Database searched** | **Platform** | **Years of coverage** | **Records** | **Records after duplicates removed** |
| --- | --- | --- | --- | --- |
| Medline ALL | Ovid | 1946 - Present | 94 | 94 |
| Embase | Embase.com | 1971 - Present | 229 | 144 |
| Web of Science Core Collection* | Web of Knowledge | 1975 - Present | 107 | 25 |
| **Total** | | | **430** | **263** |

*Science Citation Index Expanded (1975-present) ; Social Sciences Citation Index (1975-present) ; Arts & Humanities Citation Index (1975-present) ; Conference Proceedings Citation Index- Science (1990-present) ; Conference Proceedings Citation Index- Social Science & Humanities (1990-present) ; Emerging Sources Citation Index (2005-present)

No other database limits were used than those specified in the search strategies

**medline**

#1

((((HSIL) ADJ3 (vulva*)) OR VHSIL OR ((vulva*) ADJ3 (intraepithelial* OR intra-epithelial*) ADJ3 (neoplas* OR lesion*)) OR VIN).ab,ti,kf.) NOT (Case Reports / OR (case-report*).ti.)

#2

(Imiquimod / OR (imiquimod OR aldara).ab,ti,kf.)

#3

(Vulvectomy / OR Vulvar Neoplasms /su OR (excision* OR resect* OR (surg* ADJ3 remov*) OR vulvectom*).ab,ti,kf.)

#4

(Lasers/ OR Laser Therapy/ OR Laser Coagulation/ OR (laser*).ab,ti,kf.)

#5

1 AND ((2 AND 3) OR (2 AND 4) OR (3 AND 4))

**embase**

#1

('vulvar high-grade squamous intraepithelial lesion'/exp OR 'vulvar intraepithelial neoplasia'/de OR (((HSIL) NEAR/3 (vulva*)) OR VHSIL OR ((vulva*) NEAR/3 (intraepithelial* OR intra-epithelial*) NEAR/3 (neoplas* OR lesion*)) OR VIN):ab,ti,kw) NOT ([conference abstract]/lim AND [2000-2022]/py) NOT ('case report'/de OR (case-report*):ti)

#2

(imiquimod/de OR (imiquimod OR aldara):Ab,ti,kw)

#3

(excision/de OR 'local excision'/de OR 'burn excision'/de OR vulvectomy/de OR 'cancer surgery'/de OR 'vulva tumor'/exp/dm_su OR (excision* OR resect* OR (surg* NEAR/3 remov*) OR vulvectom*):Ab,ti,kw)

#4

('laser vaporization'/de OR laser/exp OR 'laser therapy'/de OR 'laser surgery'/exp OR 'laser ablation system'/de OR 'laser coagulation'/de OR (laser*):ab,ti,kw)

#5

#1 AND ((#2 AND #3) OR (#2 AND #4) OR (#3 AND #4))

**Web of science**

#1

TS=((((HSIL) NEAR/2 (vulva*)) OR VHSIL OR ((vulva*) NEAR/2 (intraepithelial* OR intra-epithelial*) NEAR/2 (neoplas* OR lesion*)) OR VIN)) NOT TI=((case-report*)) AND DT=(article)

#2

TS=((imiquimod OR aldara))

#3

TS=((excision* OR resect* OR (surg* NEAR/2 remov*) OR vulvectom*))

#4

TS=((laser*))

#5

#1 AND ((#2 AND #3) OR (#2 AND #4) OR (#3 AND #4))
